# Bioluminescence Resonance Energy Transfer as a Method to Study Protein-Protein Interactions: Application to G Protein Coupled Receptor Biology

**DOI:** 10.3390/molecules24030537

**Published:** 2019-02-01

**Authors:** Chayma El Khamlichi, Flora Reverchon-Assadi, Nadège Hervouet-Coste, Lauren Blot, Eric Reiter, Séverine Morisset-Lopez

**Affiliations:** 1 Centre de Biophysique Moléculaire, CNRS, UPR 4301, University of Orléans and INSERM, 45071 Orléans, France; chayma.el-khamlichi@cnrs-orleans.fr (C.E.K.); flora.reverchon@cnrs-orleans.fr (F.R.-A.); nadege.hervouet@cnrs-orleans.fr (N.H.-C.); lauren.blot@cnrs-orleans.fr (L.B.); 2 PRC, INRA, CNRS, Université François Rabelais-Tours, 37380 Nouzilly, France; eric.reiter@inra.fr

**Keywords:** BRET, receptor-protein interactions, G protein-coupled receptors, GPCR signaling, GPCR-interacting proteins, drug discovery, screening

## Abstract

The bioluminescence resonance energy transfer (BRET) approach involves resonance energy transfer between a light-emitting enzyme and fluorescent acceptors. The major advantage of this technique over biochemical methods is that protein-protein interactions (PPI) can be monitored without disrupting the natural environment, frequently altered by detergents and membrane preparations. Thus, it is considered as one of the most versatile technique for studying molecular interactions in living cells at “physiological” expression levels. BRET analysis has been applied to study many transmembrane receptor classes including G-protein coupled receptors (GPCR). It is well established that these receptors may function as dimeric/oligomeric forms and interact with multiple effectors to transduce the signal. Therefore, they are considered as attractive targets to identify PPI modulators. In this review, we present an overview of the different BRET systems developed up to now and their relevance to identify inhibitors/modulators of protein–protein interaction. Then, we introduce the different classes of agents that have been recently developed to target PPI, and provide some examples illustrating the use of BRET-based assays to identify and characterize innovative PPI modulators in the field of GPCRs biology. Finally, we discuss the main advantages and the limits of BRET approach to characterize PPI modulators.

## 1. Introduction

Among therapeutic targets, GPCRs represent 40% of prescription drugs and small molecules and biologics are continually being developed to modulate their activity [1]. These druggable targets regulate several important biological functions. As a consequence of mutations in GPCRs, dysregulation in their activities are involved in numerous diseases and disorders, such as allergies, cardiovascular failures, cancers or neurological and neurodegenerative diseases. Over the past 15 years, various approaches including RET methods (FRET, BRET, TR-FRET), biochemical methods, proteomic approaches and yeast two-hybrid screens have been successfully used to identify and study protein networks interacting with GPCRs. It is now well established that these receptors function as dimeric/oligomeric forms, either ligand-driven or constitutive and can interact with multiple effectors to regulate signaling and trafficking. The signaling networks associated with GPCRs are essential in nearly all biological processes such as proliferation, differentiation, migration or cell survival. Dysregulation in these protein complexes can be the cause and/or the results of abnormal downstream signaling and, as a consequence the molecular origin of pathophysiological states [2]. For this reason, PPIs can be considered as potential and innovative targets for therapeutic intervention. From the analysis of several hundreds of transient PPIs, it was shown that the minimum of protein surface that must be engaged to form a functional complex is in the order of 900 Å^2^ (around 500 Å^2^ provided by each partner) [3]. Considering the relatively large surfaces typically involved in protein-protein pairing, the identification of tools to address protein interaction targets has historically been considered as a challenge. They are frequently deemed as ‘undruggable targets’ due to their highly dynamic structures with shallow binding surface [4]. Yet, studies have shown that PPIs are not necessarily flat and are mediated by hot spots residues, where specific localized interactions must contribute to binding specificity as well as binding affinity [5]. Thus, increasing interest in this field leads to the identification of small-molecule inhibitors of protein-protein interactions (SMPPI) as novel therapeutic agents [6]. Other strategies to manipulate protein-protein interaction are based on the use of biologics tools. Interestingly, interfering peptides, antibodies, nanobodies as well as emerging oligonucleotide therapeutics such as aptamers have also been explored as new therapeutic agents [7,8]. However, this field of research remain very challenging. This is partly due to the difficulty to develop assays enabling the detection of protein-protein interaction and adaptable to high throughput screening to identify disruptors/modulators of such interactions. In this review, we first describe the different BRET systems that have been developed up to now, highlighting their advantages and their limits to study PPI. We then introduce different class of pharmacological agents known to modulate PPI.

## 2. BRET: An Overview of Developed Systems

### 2.1. Principle of the Method

**Figure 1:**
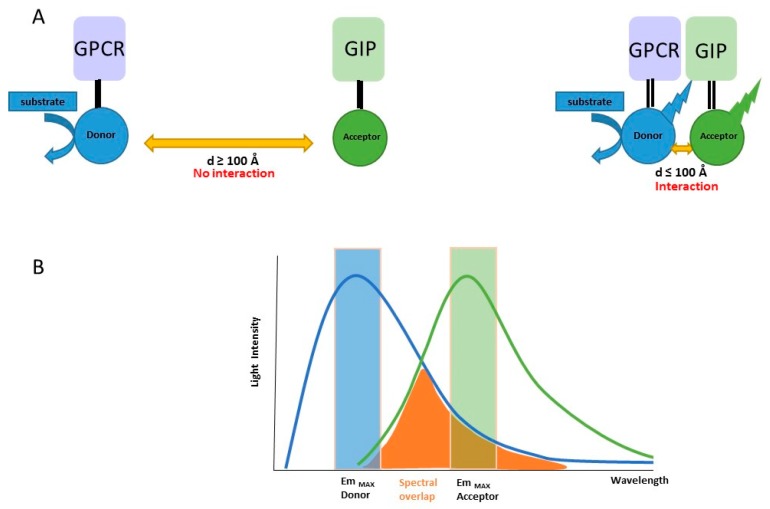
Bioluminescence Resonance Energy Transfer method. (**A**) BRET is suitable to detect the interaction of GPCR with GPCR-interacting proteins (GIP) in living cells when the distance between the two partners is <100 Å. (**B**) Basic properties of donor and acceptor molecules in order to gain BRET: the emission spectrum of the donor should overlap with the excitation spectrum of the acceptor.

Bioluminescence Resonance Energy Transfer (BRET) was originally observed in marine animals such as the sea pansy *Renilla reniformis* and the jellyfish *Aequoria victoria*. Then, BRET was applied to study protein-protein interactions in living cells. Non radiative energy is transferred from a luminescent donor (Renilla Luciferase; Rluc) to a fluorescent acceptor protein, usually the Yellow Fluorescent Protein (YFP). When studying the interaction between two proteins, BRET partners are created by expressing specifically engineered cDNAs from one protein fused to the donor and the other to the acceptor (Figure 1). After coexpression of donor and acceptor proteins in cells and upon addition of a cell-permeable substrate like coelenterazine (CLZN), luminescent signals are measured at 480 nm (RLuc light emission) and 530 nm (YFP light emission). When the donor is in close proximity, or interacts with the acceptor, the energy resulting from catalytic degradation of the coelenterazine derivative substrate is transferred from the luciferase to the YFP, therefore induces an additional light emission at 530 nm. The distance range at which energy transfer can occur is of the same order of magnitude as conventional protein dimensions (<100 Å), making BRET particularly adapted to study protein-protein interactions. Several factors can influence the intensity of BRET signal [9,10,11]. First is the distance between the two BRET partners, which is inversely proportional to transfer signal intensity: the BRET signal decreases when the distance between donor and acceptor molecules increases from 10 to 100 Å. Second is the overlap of the emission spectrum of the donor with the excitation spectrum of the acceptor (Figure 1). Third is the relative orientation of BRET pairs due to the dipole-dipole nature of resonance energy transfer mechanism. Fourth is the donor/acceptor ratio, illustrated in the BRET donor saturation assays where a fixed amount of donor molecules is co-expressed with increasing acceptor concentrations [12]. In the case of specific interactions, BRET signal increases in a hyperbolic manner and reaches a plateau when all donors have interacted with acceptor molecules. However, the use of high protein levels in BRET experiments increases the risk of detecting nonspecific signals due to random interactions. This non-specific BRET signal is generally weak and linearly increases with increasing acceptor concentrations in BRET donor saturation assays. Therefore, the detection of a BRET signal does not necessary reflect a specific PPI interaction, conversely, the absence of BRET signal between two proteins does no more mean that the tagged proteins do not interact with each other.

### 2.2. Developed BRET Systems

In the last decade, a large number of BRET systems were reported for biological applications in order to increase the magnitude and/or stabilization of BRET signal (Table 1). Much improvement has been made by changing luminescent donor, fluorescent acceptor as well as the substrate. The main advantages and drawbacks of these different BRET versions have been listed in Table 1 and would be taken into consideration depending on the objective to be achieved.

#### 2.2.1. BRET 1

BRET was described in 1999 [32] as an attractive alternative method to the related fluorescence resonance energy transfer (FRET) [33] for biological applications to detect the interaction of cyanobacterial circadian clock proteins. The initial BRET system used Renilla Luciferase as donor, eYFP as acceptor and coelenterazine h (CLZh), a hydrophobic molecule that is able to permeate cell membranes. This system gave generally BRET signal with decent brightness and high substrate stability but low Föster distance (R0). One of the most critical parameter to obtain BRET signal depend on the Föster distance (R0), the intermolecular separation characterized by 50% of the maximum possible energy transfer which can be measured by any donor-pair. This parameter gives a mean of estimation the range of distance between the two protein partners where a BRET signal can be detected. Experimental data [13] showed that R0 for BRET 1 is around 4.4 nm. In addition, considering that the wide spectrum of RLuc emission which overlaps with eYFP emission reducing the signal to noise, others BRET systems have been developed to counteract these problems. First, BRET 1 signal have been enhanced by using mutants of RLuc, such as RLuc2 ou RLuc8 [34] or RLuc8.6 [18] that are significantly brighter, with faster maturation times and more stable than the native RLuc. Then others assays have also been performed with Enduren, an optimized luciferase substrate which is very stable and can be used to monitor PPI interaction for longer time (>2 h).

Improvement was also achieved by using Venus as acceptor, which has a faster maturation times and enhanced brightness compared to YFP [35], increasing donor and acceptor spectral separation and the working distance range (2.8–8.5 nm) [13] (Table 1). Then, others BRET systems have been developed for more efficient BRET imaging by using acceptors with red shift wavelengths. The use of mOrange, TagRFP or Turbo FP with respectively peak emission at 564, 584 and 635 nm wavelengths was tested using another coelenterazine analogue named coelenterazine-v, which shifts the RLuc8 emission to 515 nm, making the assay more compatible with red-shifted acceptor [18].

BRET 1 method using Gaussia Luciferase (Gluc), cloned from the marine copepod *Gaussia princeps*, which has a size similar to Rluc (around 20 kDa) but is 100-fold brigher has also been tested to increase sensitivity. However, when it is expressed in cells, Gluc is secreted, 97% of the luminescence signal emitted is detected in the supernatant, the remainder 3% being from cell-associated enzyme. As a consequence, this luciferase is not suitable for studying intracellularly PPIs neither for kinetic studies, the luminescence peaks within the first 30 s and decays very rapidly after addition of the substrate [20]. However, GLuc presents some advantages, it shows a better tolerance to buffer composition and pH compared to RLuc [21]. Thus, all these parameters should be taken in consideration when setting up a screening assay to study PPI depending on the nature and the localization of the interacting proteins to be studied.

#### 2.2.2. BRET 2

In order to increase the separation of the two emitted wavelengths between the donor and the acceptor observed in BRET 1 method and to reduce the signal/noise ratio, a coelenterazine derivate, the DeepBlue C or coelenterazine 400a was developed by Packard. After addition of this substrate, the Rluc emission peak is reached at 397 nm, which allows emission of the compatible energy acceptor GP2 (a mutant of aequora GFP). BRET2 has also been successfully applied for various drug screening [15,23,36]. However, Deepblue C induced a weak luminescence signal lasted only a few seconds before dropping, making this assay less sensitive that BRET1. In addition the necessity to increase signal detection was circumvented by increasing the protein expression level thus increasing the risk to detect random interactions rather than specific PPI.

#### 2.2.3. BRET 3

The BRET 3 system uses the most extensively exploited luciferase in biology, the firefly luciferase (FLuc) which is used in reporter gene, ATP sensor or complementation assays. This enzyme works with D-luciferin as substrate to generate a maximum emission of light at 565 nm with longer lasting brightness compared to BRET 1 and BRET 2 systems. This red-shift variant of luciferase (FLuc) can be combined with the red fluorescent acceptor dyes such as DsRed [17,22], Cy3/Cy5 [25] or quantum dot [30]. However, the overlap of the donor/acceptor emission (565 nm/580 nm), the sensitivity of Fluc to ATP level, temperature, ionic strength and its larger size (62 kDa) have limited the spread of this assay for drug screening. Yet, with its red-shift emission wavelength and its higher light output, BRET 3 presents some advantages for imaging in living animal [17].

#### 2.2.4. NanoBRET

To overcome the limitations of the luciferases described above, such as their size, stability or brightness, a novel bioluminescence system which offer a more efficient light emission with enhanced biochemical and physical characteristics has been recently described. An optimized blue-shifted luciferase named OLuc was engineered from a small luciferase subunit (19 kDa) isolated from the deep sea shrimp *Oplophorus gracilirostris* [37]. Mutagenesis of this luciferase allowed to obtain an optimized version named Nanoluciferase (NLuc) which is higher expressed and more stable than Oluc. By the development of a novel imidazopyrazinone substrate, the furimazine, NLuc produces a 150-fold higher signal which is more stable with a signal half-life multiplied by more than 4 (>2 h) compared to both FLuc and RLuc systems [38]. In addition NLuc exhibits high physical stability, retaining activity following 30 min incubation up to 55 °C or at 37 °C in culture medium for >15 h and stays active over broad pH range. Therefore, NanoLuc has been successfully applied as a genetically-encoded partner. Currently there are several NLuc protein fusion vectors allowing expression of proteins exported to the culture medium (secreted protein) or localized intracellularly in different compartments (RE, nucleus, cytoplasm) or at the cell surface. Besides to its use as luciferase reporter, in complementation assays or molecular imaging [39,40,41], NanoLuc was also successfully used as energy donor in BRET-based assays allowing development of highly sensitive biosensors. These systems were particularly developed to monitor the binding of ligand to receptors. Thus, several nanoBRET binding assays using GPCR tagged with NanoLuc in the N-terminal part of the receptor with BODIPY or TAMRA fluorescent ligands have been applied to overcome drawbacks of radioligand binding assays [42,43,44,45]. To study PPI, nanoBRET systems have also been developed with an optimal fluorescent acceptor fused to HaloTag [26]. HaloTag (HT) technology is carried out using a two-step approach which consists in the fusion of a stable HaloTag protein (33 kDa) with the protein of interest and the addition of a chloroalkane (HaloTag) ligand that bind rapidly and irreversibly to the HaloTag-fused protein. Among the HaloTag ligands tested, the highest BRET signal was achieved with a chloroalkane derivative of nonchloro TOM (NCT) dye, which has an excitation maximum at 595 nm and a peak light emission at 635 nm [26]. This BRET pair, NanoLuc/ HaloTag system allows to effectively reduce the background caused by the donor signal into the acceptor channel. The higher brightness of NanoLuc allows the detection of PPIs at low levels comparable to endogenous physiological conditions. The ability to perform such measurements at low concentrations of reporter may be particularly relevant when studying PPI in challenging cell types, such as stem cells, primary cells, or neuronal cell types, which are particularly hard to transfect. This advantage should allow in the near future to detect PPI in individual cells by microscopy imaging. In addition, nanoBRET offers the possibility to detect PPI in trans, i.e., between cells [46], a feature that could not be possible with others BRET systems [47]. Overall, NanoLuc BRET assays exhibits a higher sensitivity, an improved spectral resolution and dynamic range as well as a more stable luminescence signal compared to current BRET systems. It holds a great potential to study PPI and to identify PPI modulators. The main limitation for its use is the requirement of furimazine, an optimized synthesized substrate [38], which is a very expensive and is not generically available.

#### 2.2.5. Quantum Dot-Based BRET (QD-BRET)

Besides these systems, nanoparticules named quantum dot (Qdot, QD) have also been tested in BRET assays and applied for in vivo imaging [28]. QDs are particularly advantageous over organic dyes or fluorescent proteins because of their unique optical properties including low photobleaching, broad absorption spectra and narrow emission spectra, high quantum yield and high photochemical stability. As a consequence, Qdots have been extensively used in the development of biosensors and biomarkers assays as well as for in vitro and in vivo imaging [48]. Most QD-BRET systems have used RLuc and its variants as donor molecules with different types of quantum dots [28,48,49]. Others BRET assays using firefly luciferase as donor and QD as acceptor have also been tested [30,31]. More recently, a QD-NanoBRET system using NLuc as donor and Quantum dot705 as acceptor was successfully performed for tumor imaging [50]. In this study, QDot were used as a platform to conjugate both Nluc for molecular imaging and a cyclic peptide known to strongly bind to integrin α_v_β_3_ expressing by tumor cells for targeting the tumor. This QD-Nluc conjugate showed an excellent signal/noise signal for monitoring complex biological processes in living cells or animals systems [50]. However, to our knowledge, the use of these quantum dots as acceptor in BRET pair has never been explored in PPI screening cell-based assays probably due to their large size (2–10 nm) for labelling and the necessity to label the studied protein with quantum dot. Different strategies including streptavidin QD targeting a biotinylated antibody, cross-linking of primary or secondary antibodies, receptor ligands or recognition peptides to QDots have been explored to detect the proteins of interest [51]. However, the cumulative volume of antibodies and QDots as well as other factors such as the processing parameters or cellular toxicity of QD [52] may hinder their usefulness in studying PPIs. Considering the possibility of multiplexed sensing using QDs with different wavelengths of emission, it is conceivable they could be useful in the development of assays for simultaneous detection of multiple protein-protein interactions in living cells.

## 3. G protein Coupled Receptors Form Molecular Complexes

GPCR are among the most abundant membrane proteins in humans with more than 800 of such receptors identified up to now, and more are being discovered over time. They are found on cell surface or intracellularly and play key roles in cell signaling. GPCRs are defined by seven transmembrane (TM) helices linked by three extracellular and three intracellular loops [53,54]. The N-terminal sequence is extracellular whereas the C-terminus is intracellular, a topology that allows several exposed domains for potential protein-protein interactions. Ligands for these receptors can be numerous and differ in their nature (chemicals or proteins), size and structure. Some of them are glycoprotein hormones, pheromones, growth factors, neurotransmitters or small molecules like sucrose or calcium ions [53].They are known to interact with various proteins such as receptors or intracellular proteins to regulate their transduction, signaling and trafficking.

### 3.1. GPCR Form Dimers/Oligomers

For a long time GPCRs were thought to be monomeric entities, but results of a large amount of studies accumulated over the last twenty years show that they can form dimers or ordered oligomers [55,56]. Formation of dimers to achieve appropriate targeting of a given receptor is a commonly observed phenomenon in the field. The ability to form homo and hetero-dimers involving diverse GPCR subtypes with the same or different ligands has potentially far reaching implications, especially with regard to drug discovery. Many early studies exploited this phenomenon to identify potential GPCR dimers [57]. There are many reported cases where one of the receptors in a GPCR dimer can serve to alter the coupling or regulate the potency of the second receptor. While the dimerization of Class C GPCRs, including GABA_B_ receptors [58,59,60,61] or metabotropic glutamate receptor [62] is widely accepted, the occurrence and functional consequences of rhodopsin like Class A GPCR dimerization remains a debated issue. However, a large amount of data supports the existence of homo- and heteromers of class A GPCRs in intact cells, even in native tissues [62,63]. The effects of GPCR oligomerization on receptor pharmacology and signal transduction are well documented as well as its implication in physiology and pathophysiology [55,64,65,66]. These advances have been achieved thanks to the use of biophysical and biochemical methods. Among these methods, BRET has strongly contributed to the demonstration of this phenomenon in living cells. The pioneer study conducted by Bouvier’s laboratory demonstrated β2AR dimerization through this approach [67]. Then, the use of BRET-based assays to demonstrate homo or hetero-imerization of GPCRs has rapidly grown [68,69,70,71] to the point that they have been listed in the GPCR Oligomerization Knowledge Base http://www.gpcr-okb.org [64]. BRET is also considered as a valuable technique for the investigation of the structural basis of dimerization. For example, it was used to identify critical residues involved in the constitutive homodimerization serotonin 5-HT_4_ [72] or the M3 muscarinic acethylcholine receptor [73]. If BRET is able to monitor all kinds of interaction, however, certain concerns have to be taken into account when studying association of membrane proteins, such as GPCR. Several authors have used BRET and other methods to cast significant doubt on the existence and prevalence of class A dimers [74,75,76,77]. The analysis of donor saturation assays may be potentially miss interpreted, particularly in transient transfection system, when the expression of the energy donor has to be maintained constant. Donor expression often decreases substantially as acceptor expression increases, despite the constant amount of DNA encoding the donor molecule. In this case, it is possible to observe a hyperbolic curve in donor saturation assays, even if the interaction between membrane proteins is nonspecific leading to false interpretation of dimerization state of GPCR. Altogether, these data demonstrate that BRET is a reliable method for assessing membrane protein association, as long as donor saturation experiments have been properly controlled and interpreted cautiously.

### 3.2. GPCR Signaling through G Proteins

After activation by ligands, GPCRs activate one or more heterotrimeric G proteins by eliciting GDP/GTP exchange on α subunit. Heterotrimeric G proteins transduce ligand binding to receptors into intracellular responses, which underlie physiological responses of tissues and organisms. Although there are many gene products encoding each subunit (20α, 6β, and 12γ gene products are known), they are grouped in four main classes: G_S_, which activates adenylyl cyclase; G_i_, which inhibits adenylyl cyclase; G_q_, which activates phospholipase C; and G_12_ and G_13_ which are less described [1]. G proteins are inactive in the GDP-bound, heterotrimeric state and are activated by receptor-catalyzed guanine nucleotide exchange resulting in GTP binding to α subunit. GTP binding leads to dissociation of Gα GTP from Gβγ subunits and activation of downstream effectors by both Gα GTP and free Gβγ subunits. G protein deactivation consists in Gα subunit hydrolysis of GTP to GDP, a rate-limiting step resulting in turning off the cellular response [78]. Different BRET systems have been proposed to monitor the interaction between GPCRs and their cognate G proteins. BRET 1 or BRET 2-based assays, in which the receptor was fused to RLuc (or YFP) and Gα, Gβ or Gγ subunits was fused to GFP2 (or RLuc), were successfully applied to monitor G protein activation upon GPCR stimulation in real time [79,80]. GPRC activation was also monitored using BRET probes inserted at multiple sites in receptor G protein complexes allowing the detection of conformational changes in receptors and G proteins complexes as well as between G proteins subunits [81]. Other assays have been developed to monitor the release of free Gβγ dimers after activation of heterotrimeric G protein, which were also able to bind to freely-diffusing acceptor, the C-terminus of GPCR kinase 3 (GRK3ct). Originally, the GRK3ct fused to RLuc8 was used as donor and the free βγ dimers fused to a venus serves as acceptor [82]. This cell-based assay has been improved by the replacement of Renilla Luciferase (RLuc8) by the engineered NLuc [83]. The system has been also extended to study the activity of 14 different G proteins [83]. More recently, the recruitment of G protein to active GPCR upon agonist treatment was monitored by BRET using mini G protein probes [40]. In this study, GPCRs fused to RLuc8 was used as donor, and mini G protein (mG_s_, mG_i_, mG_q_ and mG_12_) corresponding to the four families of Gα subunits were fused to venus and served as acceptor.

### 3.3. GPCR Interact with Multiprotein Complexex

It has been known for a long time that GPCRs interact directly not only with heterotrimeric G proteins but also with various accessory proteins called GPCR-interacting proteins (GIP). These proteins are implicated in GPCRs targeting to specific subcellular compartments, in the assembling into large functional complexes called receptosomes or in the termination of the signal [84,85,86]. The first proteins identified as GIP were the GPCR kinases (GRKs) [87] and arrestins [1,88]. Following activation, GPCRs are phosphorylated by G protein-coupled receptor kinases, and subsequently recruit cytosolic β-arrestins. β-arrestins binding uncouple the receptors from G proteins and desensitizes G protein-mediated signaling [89]. However, it has been shown that, in addition to their desensitizing actions, β-arrestins also serve as multifunctional adaptors and signal transducers, linking the receptors, in an activation-dependent manner, to a growing list of endocytic and signaling molecules. Over the last twenty years, a large number of GIPs have been described such as small GTP binding proteins Ras, Rab, Rho and ARF, that have typically been viewed as a downstream consequence of heterotrimeric G protein activation or the regulators of G protein signaling (RGSs) [84,90,91]. There is also a family of proteins, the PDZ Domain–containing Proteins which have been described as GIPs and recognize at the C-termini of GPCRs, a PDZ (PSD95/Dlg/ZO-1) ligand typically composed of three or four amino acid residues. There is increasing evidence showing that the interaction of GPCRs with PDZ proteins are important to regulate GPCR targeting, function and pharmacology [92]. Therefore, targeting GPCR/GIP interactions may be considered as a novel approach for therapeutic intervention. Indeed identification of specific disrupters/stabilizers of a given GPCR/GIP interaction that do not affect the interaction of the same GPCR with other GIPs might be considered as attractive strategy to “bias” GPCR signals by preferentially stabilizing an active conformational state of the receptor. In the last decade, this concept has been applied and raised to the development and characterization of various biased ligands that have great therapeutic potential in a number of indications, such as pain, cardiovascular diseases or cancers [93,94]. These ligands are considered as optimized therapeutics with improved efficacy and/or reduced side-effect profiles. In this context, identification of agents able to stabilize subset of active conformational states by targeting GPCRs/GIP offer exciting opportunities to modulate GPCR signaling. BRET has been extensively applied to monitor GPCR/GIP either statically or dynamically, in real time in living cells. The pioneer studies have exploited BRET to study the interaction between GPCRs and β-arrestins. After the fusion of both the receptor and β-arrestins to energy donor and acceptor, BRET signal is detected after receptor activation as soon as the transconformational change of GPCR allows the recruitment of β-arrestins. This assay has been reported for the association of β-arrestins with various GPCRs [68]. Then BRET was applied to study the dynamic interaction of GPCRs with others GIP such as CdK5 [95] or neurofibromin [96].

## 4. Tools to Target GPCR/Protein Interaction

**Figure 2:**
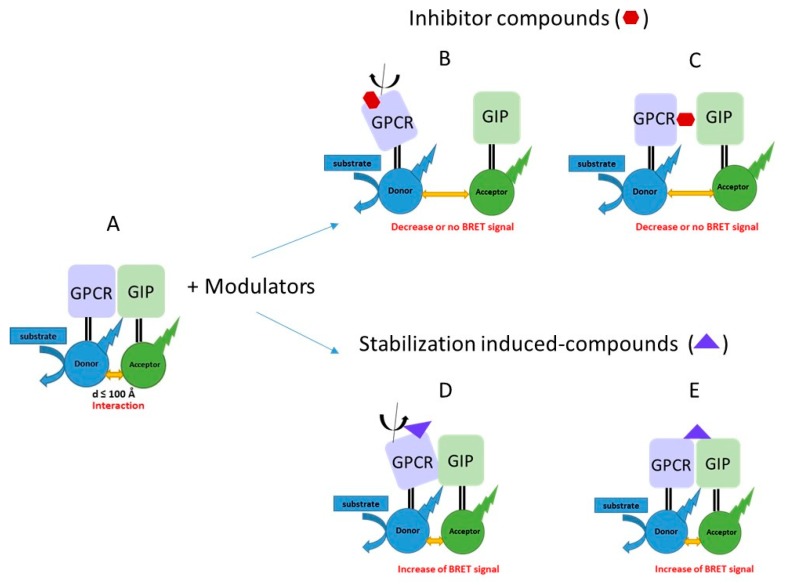
Modulators can induce PPI inhibition or stabilization. GPCR/GIP interaction can be detected by BRET (**A**). Modulators for GPCR/GIP interaction may function using orthosteric (binding at the protein-protein interface; (**C**,**E**) and allosteric (through conformational change of one protein in the complex; (**B**,**D**) mechanisms to lead to PPI inhibition or stabilization.

Protein-protein interactions are central in most biological processes and, therefore, represent a vast class of therapeutic targets. Depending on the method used to study PPIs, the resource available, the nature of the targets or the cellular localization of the interaction, different classes of PPI modulators have been developed. Although the major effort on modulating GPCRs signaling has initially been focused on the identification of chemical compounds, others strategies based on the development of interfering peptides, antibody fragments or RNA aptamers have recently emerged. BRET is particularly useful to identify modulators that can disrupt or stabilize protein complexes either by binding at the protein-protein interface (orthosteric mechanisms) or through allosteric mechanisms by inducing conformational changes of one of the partner (Figure 2).

### 4.1. Small Molecules Libraries

Because the great majority of PPIs takes place inside the cell, the early efforts of PPI modulators development focused on small molecules, which may have the ability to passively diffuse across the cell membrane and can be administrated orally for therapeutic application. In addition HTS assays may be applied to identify small molecules PPI inhibitors/modulators. The choice of the chemical library to be used is of crucial importance in drug discovery. Historically, the first strategy to obtain chemical library was based on the development of a large collection compounds constructed in a combinatorial fashion with strong chemical diversity, therefore, being used against a large diversity of targets. Then, the quality of library has been improved over the years by the application of selection filters driven by the physicochemical and pharmacokinetics properties of medicine already approved or in clinical phase and by the results obtained in previous screens to remove compounds known to be frequent hits. More recently, alternative chemical libraries have been developed in order to obtain libraries focused on a particular target, family of proteins or mechanism of action [97]. Thus, strategies have been used to rationally design focused libraries targeting protein-protein interactions (PPIs) that predominantly rely on designing scaffolds for accurately mimicking protein secondary structure, for targeting hot-spots. Due to the favourable pharmacokinetic properties of many small organic molecules (<600 molecular weight), the design and synthesis, almost forty compounds targeting PPIs reached clinical development in different therapeutically area, such as of cancer, cardiovascular or asthma [98].

More recently, the use of a collection of synthetic macrocycles scaffolds with some based on natural products have also emerged as an exciting new avenue for modulation of target PPI. With molecular mass typically ranging from 500 to 2000 Da, macrocycles are 3–5 more times larger than conventional small molecule drugs allowing binding to large surfaces that are similar in size to those of antibodies and native PPI interfaces [99]. The use of such compounds to target protein interfaces have been successfully explored [100]. Although these molecules retain some interesting properties such as a metabolic stability and lack of immunogenicity, they unfortunately possess some limitations for clinical applications. They are difficult to rationally synthesize by conventional approaches, most of them are not permeable to cell membrane, which limit their use to target intracellular PPIs [100].

Most efforts on targeting GPCRs signaling remain focused on the identification of small molecules acting on orthosteric and allosteric sites at the extracellular surface of GPCRs. BRET-based assays are extensively used to study the effects of such ligands on GPCR/GIP interaction [101]. However, in the past few years, studies have also revealed that some small molecules can modulate receptor function by interacting with the intracellular side of GPCRs. It was shown that the interaction surface of these intracellular ligands overlap to some extend with binding sites of signaling molecules such as Gα_S_ and β arrestins [102]. Therefore, by precluding the interaction of the receptor with its interacting proteins, these intracellular ligands act as innovative PPI inhibitors.

### 4.2. Peptides/Peptidomimetics

Peptides are considered as excellent approach for the development of PPI modulators. Peptides-based modulators are designed or screened to selectively bind distinct sites of specific proteins, thereby increasing efficacy, specificity and selectivity and decreasing the risk of triggering off-target effects. However, the use of such peptide in therapeutics may involve rational chemical modification to increase their stability or need conjugation with cell-penetrating sequence (like Tat). The use of Tat-conjugated peptides has clearly illustrated the therapeutic interest of these peptides that compete for specific GPCR/GIP interactions. The first study in the GPCR field showed that a Tat peptide, encompassing a motif located in the i3 loop of 5-HT_2C_ receptor and able to disrupt 5-HT_2C_R/PTEN complex, suppress behavioural responses induced by drugs of abuse, highlighting the therapeutic potential of peptides [103]. Disruption of another complex between 5-HT_2A_ receptor and PSD 95 with a Tat-conjugated peptide was shown to induce an antihyperalgesic effect and to strongly enhance the efficacy of selective serotonin reuptake inhibitor (SSRI) efficacy used for the treatment of neuropathic pain [104]. More recently, BRET was successfully applied to identify a tat-conjugated peptide able to disrupt the interaction of 5-HT_6_ receptor with neurofibromin. This study suggest that 5HT_6_R/neurofibromin complex might contribute to the constitutive activity of the receptor on G_s_ signaling and that the loss of this complex in neurofibromatosis type 1 might contribute to the appearance of cognitive deficit observed in patient [96]. The use of Tat-conjugated peptides may present some limitations which are their high sensitivity to proteases in vivo, their often poor solubility or their hydrophobia that consequently may affect their penetration inside the cell when targeting intracellular PPIs. To overcome these limitations other biopolymer mimetics, such as peptoids, first reported by Simon et al. [105], are the most documented examples of peptidomimetics compounds. Peptoids are formed of oligomers of N-substituted glycine (NSG) units, which are ideal for combinatorial approaches to drug discovery. Large libraries can be easily synthesized from readily available primary amines. Peptoids provide some advantages over natural peptides, including an enhanced stability toward proteolysis, a better cell penetration, a lower immunogenicity and a reduced sensitivity to denaturation induced by solvent, temperature or chemicals denaturant. Indeed, secondary structures in peptoids do not involve hydrogen binding [106]. Thus, the use of peptoids libraries have already been applied in drug discovery, in particular in the field of GPCR [107]. To study PPI involving transmembrane proteins such as GPCRs, others categories of peptidomimetics such as transmembrane peptides have also been developed and may be considered as promising agents for biomedical applications in particular to target PPI in membranes [108]. Moreover, pepducins, cell permeable peptides derived from intracellular loops of the receptors have also been successfully utilized to modulate receptor signaling from inside. These biologics can access intracellular binding sites of cognate GPCRs where they can prevent or stabilize the recruitment of effector proteins stabilizing unique receptor conformations, which in turn can elicit pharmacological signaling profiles [109].

### 4.3. Antibodies and Nanobodies

Antibodies provide excellent affinity and specificity for their cognate targets. Since the first antibody approved by the FDA in 1997, they have become increasingly important for the treatment of human diseases. To date, around 60 therapeutic antibodies are on the market. Despite the success of monoclonal therapeutic antibodies, to date there is only two FDA approved GPCR-directed antibody: erenumab, an antibody that inhibits the calcitonin gene-related peptide (CGRP) receptor, used for the treatment of chronic migraine [110] and mogamulizumab approved for the treatment of cutaneous T cell lymphoma that targets CCR4 [111]. There are several reasons for the delay in the development of anti-GPCR antibodies, including the time and expense of production of monoclonal antibodies, tendency to elicit immune responses, a limited metabolic sta0bility, bioavailability and a low cell permeability. However, recent advances in the development of antibody have shown some promise to overcome these hurdles. Therefore, alternate engineered antibodies structures with fragment antibodies have been developed. Recently, a fragment antibody derived from the monoclonal antibody, termed mAb16 was shown to promote the stabilization of GPCR/G-protein complexes [112]. In addition, the discovery of nanobodies (VHH) produced in camelids has opened over the last decade new ways for targeting these receptors. Compared with conventional antibodies, nanobodies (VHH) are small (~12 kDa) and lack light chain. It was thought that the absence of light chain known to increase the diversity of the repertoire of conventional IgG would induce a loss in the diversity of VHH repertoire. It turned out not to be the case, most likely because VHH have increased lengths in their hypervariable regions CDR1 and CDR3; generate more varied conformations and present large surface area for interaction with the antigen; all features that compensate the relative limitation of their repertoire [113]. VHH, thanks to their convex shape, preferentially recognize concave antigens whereas conventional IgG paratopes are flatter and recognize flat or convex antigens [114,115]. It is this feature which makes it a particularly interesting tool for the targeting of buried epitopes such as those found in GPCRs. In addition, VHH have particular properties compared to conventional antibodies. Their monomeric structure facilitates cloning and expression. They are also poorly immunogenic, possess high stability and are resistant to extreme physicochemical conditions (temperature, pH) [116,117]. Finally, their small size provides them a better cellular penetration. Studies have reported that some VHH have the ability to pass the blood-brain barrier [118,119], this property probably depends on their sequence, 2D and 3D structures. Therefore, one of the challenges for the coming years will be the search for strategies / tools to increase their intra-tissue delivery. Other limitations will also have to be overcome in the case of therapeutic applications such as their short life after systemic administration (30 min) compared to classic IgG (15 days). Smit’s group identified the first VHH targeting a GPCR, the chemokine receptor CXCR4. These VHH act as antagonists and block the binding of the CXCL12 chemokine to the receptor [120]. Other nanobodies acting as antagonists have been described in the case of CXCR2 receptors [121] or CXCR7 [122]. In the case of the β2 adrenergic receptor, intrabodies capable of stabilizing different active or inactive receptor conformations have been identified. By binding to epitopes located at intracellular regions of the receptor, these intrabodies act as positive or negative allosteric modulators of the receptor [123,124]. Recently, other potent and selective mGlu2 glutamatergic receptor nanobodies DN10 and DN13 have been identified and act as positive allosteric modulators: they bind to the extracellular portion of the mGlu2 receptor homodimers and stabilize its active form [125]. Altogether these studies show the potential of nanobodies to stabilize or disrupt GPCR/G protein complexes and as consequence to control receptor activity.

### 4.4. Nuclei Acid Aptamers

An alternative to antibodies is the use of aptamers which present a promising modality for addressing PPIs. Aptamers are non-natural oligonucleotide, ARN or ADN molecules that show the ability to bind to specific target molecules. Given their high affinity and specificity, low immunogenicity and ease to be synthesized, aptamers have been under development for more than two decades. Aptamers are generated by an in vitro selection process referred to as SELEX (systematic evolution of ligands by exponential enrichment) for the selection and characterization of aptamers against target molecule. From a random aptamers library (10^13^–10^15^), several rounds of selection are performed to get primary hits, aptamers having very high-affinity to target [126]. Then, the selected aptamers are minimized to reduce their length and facilitate efficient manufacture at large scale. This step allows the identification of the minimal element within the hit aptamer sequence that bind the target and keeps the desired in vitro functionality. Subsequently, chemical modifications to the minimized aptamers are introduced to improve their potency, decrease their nuclease sensitivity and increase their residence time in vivo. Aptamers have been widely used in medical for diagnostic and therapeutic purposes [127]. In 2004, Macugen^®^, also known as pegaptanib, that consists in a 28 long single stranded RNA and bind to vascular endothelial growth factor (VEGF) was the first FDA aptamers to be approved for the treatment of age-related macular degeneration [128]. Several aptamers are now in clinical development [127]. Recently, RNA aptamers have also emerged as an interesting approach to target transducers downstream of GPCR such as β-arrestin [129]. Interestingly, by using SELEX technology, Lefkowitz’s group found aptamers that bind to intracellular surface of β2 adrenergic receptor and display the ability to stabilize active, inactive and ligand-specific conformations [130]. Some of them are able to inhibit agonist-induced cAMP accumulation. It was supposed that the inhibition of AC coupling induced by aptamers was mediated by their binding to intracellular region of the receptor, and as a consequence, preclude the recruitment of G protein to GPCR. Although this study did not measure the direct influence of aptamers on receptor—G protein complex, it is likely that these aptamers act as allosteric PPI modulators. Overall, aptamers are emerging as versatile and promising tools to modulate GPCR signaling.

## 5. Discussion

**Figure 3:**
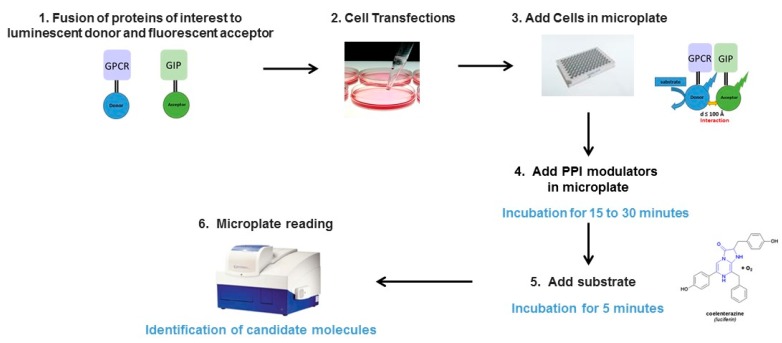
Schematic representation of high throughput screen using genetically encodable BRET-based biosensors.

Current approaches for monitoring PPIs use two hybrid systems, split reporter protein complementation and reconstitution or RET-based assays such as TR-FRET, FRET and BRET. BRET experiments, like FRET requires the addition of bioluminescent or florescent tag, generating fusions proteins, which may affect their transport between cell compartments, their post-translational modification status, their addressing, their biological properties and their degradation. Ideally, receptor-proteins interactions should be studied with native state proteins. However, BRET is certainly the best suitable method to assess PPI in their natural environment allowing applications both in vitro in living cells and in vivo in living animals. The main advantage of BRET is that it does not require extrinsic excitation by a light source, thus bypassing the concomitant excitation of the acceptor, the photobleaching of the donor as well as cell autofluorescence. Therefore, due to lack of exogenous optical excitation in BRET experiments, the background at the acceptor signal is low and all the light emitted by the acceptor protein would result from the non-radiative resonance energy transfer, leading to a BRET signal/noise ratio 10 fold higher than those obtained in FRET-based assays. BRET approaches permit the use of 40-fold less amount of protein to reach the same signal level as FRET, demonstrating the method sensitivity. In addition BRET assays are performed in homogeneous assays and do not require additional washing to separate complexes from free proteins, known to considerably limit the robustness and high throughput of the assays. Compared to the luciferase protein complementation assays (Luc-PCAs), BRET is a more sensitive method, split luciferases after functional recovery display only 20–50% of the activity of corresponding intact luciferase. In addition, false-positive signal as well as false-negative signal can be detected resulting respectively from non-specific interaction between the split fragments or from misfolding of the protein obtained after complementation. Therefore, BRET provides several advantages as it can be applied to monitor interaction (1) between various kinds of proteins: soluble or transmembrane (2) at different subcellular levels: in the nucleus, cytoplasm or plasma membranes as well as extracellularly (3) in living cells respecting the environment of the protein complexes (4) in real-time to follow dynamic interactions (5) in 96 or 384 wells plates, formats adapted to screening assays (Figure 3). In addition, BRET can also allow the detection of conformational modulators of the targeted complex. Indeed, BRET method being dependent on the relative orientation of the dipole moment of the donor and acceptor, is also suitable to detect tiny conformation changes within the complex leading to an increase or a decrease in BRET signal. However, certain concerns have to be taken into account when setting up a BRET-based screening assays to identify PPI modulators. As underlined before, different parameters can modulate the signal intensity between BRET pairs. In particular, considering the well-known donor saturation assays, the expression of both studied partners would result in an ideal window for PPI monitoring to prevent the detection of too weak BRET signal. Conversely, an excess of BRET signal, when acceptor molecules are over-expressed, may increase the risk of titration of PPI inhibitors/ modulators [10]. To avoid this last concern, the number of cells must be reduced to the minimum, while still obtaining a reliable BRET signal. Thus, in order to minimize the variability of results due to cell transfection or number of cells, the use of the same batch of biological material, namely, stable cells lines co-expressing donor and acceptor molecules would be ideal for drugs screening. BRET signal being dependent on fluorescence and luminescence parameters, fluorescent or coloured compounds as well as phenol red supplement medium may interfere with the signal and can give rise to false positive or false negative hits. In order to confirm BRET screening results, additional controls using non-related tagged protein as well as others methods allowing PPI monitoring must be applied. Despite of these limitations, BRET has already been successfully applied in high-throughput screening assays to detect modulators of PPI [10,12,23,47].

This review aims at providing emerging paradigms of modulating GPCR activity and function by identification of small molecule ligands, peptides aptamers or antibodies targeting receptor-protein interaction. Among the different methods available to study PPI, BRET offers several advantages to study GPCR biology. Up to now, BRET has been successfully applied to assess ligand-receptor, receptor-GIP or receptor-receptor complexes in living cells, making possible the monitoring of these interactions in real time. Most studies used BRET as biosensors to identify and characterize ligands that bind to the orthosteric or allosteric sites of GPCR, and as a consequence induce conformational changes within the receptor that promote or inhibit the binding to a GIP (e.g., a heterotrimeric G protein, β-arrestin) at the cytoplasmic side. However, to our knowledge, very few studies reported the use of BRET to search for modulators/ inhibitors able to disrupt/stabilize interaction by binding at the interface of complexes. We anticipate that exploiting the BRET technology has the potential to improve the discovery of PPI modulators able to modify and/or “bias” GPCR signaling, which will contribute to advances in the GPCR field.

## Figures and Tables

**Table 1:** Uses, advantages and drawbacks of different BRET systems.

Name *	Donor	λ_em_^§^	Acceptor	λ_em_^§^	Substrate	Advantages	Drawbacks	Refs
BRET 1	RLuc/RLuc8	480	eYFP	530	CLZN h	Monitor PPI at endogenous expression levels of protein RLuc8 more stable than RLuc	Sensitive to solvent polarity, serum and pH	[13,14]
BRET 1 ^1.1^	RLuc/RLuc8	480	Venus	530	CLZN h	Venus has faster and more efficient maturation compared to YFP Working distance range increased (2.7–8.3 nm) compared to BRET 1 (2.2–6.6 nm)		[13,15]
BRET 1 ^2^	RLuc	480	eYFP	530	Enduren	Monitoring of PPI several hours in real-time under near-physiological conditions	Requires expensive Enduren	[15,16]
BRET 1 ^3^	RLuc8	480	mOrange	564	CLZN h	Application for BRET imaging Wide spectral separation Δλ: 84 nm	mOrange: slow maturation processes (t_1/2_: 2 h)	[17]
BRET 1 ^3.1^	RLuc8	515	mOrange	564	CLZN v	CLZN v increases the spectral overlap between donor emission and acceptor excitation	Low spectral separation Δλ: 50 nm	[18]
BRET 1 ^4.1^	RLuc8	515	TagRFP	584	CLZN v		Low spectral separation Δλ: 70 nm	[18]
BRET 1 ^5^	RLuc8.6	535	TagRFP	584	CLZN h	Increased stability and enhanced enzymatic activity of RLuc8.6 compared to RLuc8	Low spectral separation Δλ: 50 nm	[18]
BRET 1 ^6^	RLuc8.6	535	TurboFP	635	CLZN h	High spectral separation Δλ: 100 nm Application for BRET in living animals		[18]
BRET 1 ^7^	Gluc	470	eYFP	530	CLZN h	Gluc smaller and brighter luciferase	Glu activity depends on pH and NaCl concentration Secreted luciferase	[19,20]
BRET 1 ^7.1^	hGluc	470	TdTomato	580	CLZN h	Large spectral separation compared to Gluc/eYFP pair Δλ: 110 nm High tolerance toward the solution components (serum) and pH.	TdTomato: slow maturation processes compared to GFP Low stokes shift	[21]
BRET 1 ^7.2^	hGluc	470	DsRed	583	CLZN h	Large spectral separation: Δλ: 110	DsRed: slow maturation processes, fluorescent intensity lower compared to GFP	[22]
BRET 2	RLuc	395	GFP2	510	DeepBlueC	Large spectral separation: Δλ 115 for BRET2 vs. 50 for BRET1 ^1^	DeepBlue C: weak and short lasting light emission Necessity high expression of BRET partners	[23]
BRET 2	RLuc2	420	GFP2	510	DeepBlueC	Working distance range increased (3.8–11.5 nm) compared to BRET 1 (2.2–6.6 nm)		[13]
BRET 2	RLucM/RLuc8	400	GFP2	510	DeepBlueC	RLuc8 increased stability and even higher quantum yield BRET signal 30 fold higher than RLuc/GFP2 pair Application for BRET in single live cells and living animals		[24]
BRET 3	FLuc	565	DsRed	583	D luciferin	DsRed: high photostability and resistance to pH; Application for in vivo imaging	Overlap of donor/acceptor emission Low signal/noise	[17,22]
BRET 3	FLuc	565	Cy3/Cy3.5	570/596	D luciferin		Overlap of donor/acceptor emission Low signal/noise	[25]
NanoBRET	Nluc	462	haloTag	618	Furimazine	NanoLuc is 100 fold brighter than RLuc. Furimazine permits longer observation (2 h compared to 25 min with coelenterazine)	Not red shifted version available Requires expensive Furimazine	[26]
NanoBRET	Nluc	462	Venus DsRed	535	Furimazine	Improved sensitivity and dynamic range Used as biosensor Single cell BRET imaging	Not red shifted version available Requires expensive Furimazine	[26]
QD-BRET ^1^	RLuc	480	Qdot	620	CLZN h	Used as biosensor Larger stokes shift Resistance to photobeaching Strong fluorescence		[27]
QD-BRET ^2^	RLuc8	480	Qdot	655	CLZN h	Real time in vivo imaging	Size of Qdot	[28,29]
QD-BRET ^3^	FLuc	565	Qdot	613/628 675	CLZN h	Working distance range increased	Problem for Coupling to proteins; cellular toxicity	[30,31]

* Authors gave name for each system described. However, as no standard nomenclature has ever been established, these names are not absolute or exclusive, for reference only. **^§^** Peak wavelength in nm.
